# Exertional Rhabdomyolysis and Ultra-Trail Races: A Systematic Review Highlighting the Significant Impact of Eccentric Load

**DOI:** 10.3390/muscles3030022

**Published:** 2024-08-13

**Authors:** Miguel Lecina, Carlos Castellar-Otín, Alejandro García-Giménez, Francisco Pradas

**Affiliations:** ENFYRED Research Group, Faculty of Health and Sports, University of Zaragoza, 22002 Huesca, Spain; castella@unizar.es (C.C.-O.); alejandro.garcia@unizar.es (A.G.-G.); franprad@unizar.es (F.P.)

**Keywords:** exertional rhabdomyolysis, muscle damage, blood biomarkers, ultra-trail race, eccentric load

## Abstract

Exertional rhabdomyolysis (ER) is a condition where muscle breakdown occurs after intense and unaccustomed exercise in healthy individuals. It is characterized by muscle pain, weakness, and myoglobinuria, potentially leading to acute kidney injury and worsening the patients’ prognosis. Ultra-trail races (UT) necessitate high energy and extreme muscular exertion, which can result in significant muscle breakdown, leading to ER and elevated biomarkers such as creatine kinase (CK) and lactate dehydrogenase (LDH). These races involve longer durations and both uphill and downhill elevations, with the latter causing more muscle damage. This systematic review aims to analyse the effect of downhill elevation (at least 1000 m) in UT on muscle and liver damage biomarkers. We conducted a systematic review of four electronic databases (Pubmed, Web of Science, Scopus, and Sportdiscus) based on PRISMA guidelines for systematic reviews. We included a total of 15 articles out of 6670 published between January 2005 and March 2024. The total population sample included 348 subjects, comprising 294 men (84.48%) and 54 women (15.52%) with a mean age of 39.82 ± 6.89 years. Only one subject (0.28%) was diagnosed with ER. The median increase in CK post vs. pre was 5370.63 ± 7289.71%, LDH post vs. pre was 311.20 ± 164.4%, and liver damage biomarkers aspartate transaminase (AST) and alanine aminotransferase (ALT) obtained mean increases of 1009.94 ± 743.97% and 207.02 ± 92.84%, respectively. No liver injury cases were reported. These findings suggest that ER is often misdiagnosed in UT and may result in acute kidney injury under certain circumstances. Therefore, it is crucial to define and prepare the characteristics required for ultra runners to safely participate in these extreme races.

## 1. Introduction

Ultra-endurance sports are defined as strenuous exercises that involve either a distance greater than 42.195 km or durations exceeding 6 h [1]. Examples of such events include Ironman, triathlon, ultramarathon, or ultra-trail races (UT) among others [2]. These races demand extreme effort from participants and are highly taxing on their bodies[3]. Over the years, the popularity of these races has increased significantly [4], making them one of the sought-after sports for non-professional runners [5]. In the United States alone, UT has more than 9.1 million participants [6], and the number of races worldwide has doubled in the last five years [7,8]. Mountain running, sky running, and trail running are all disciplines that share a common background and take place in natural [9] environments featuring off-road pavements, minimal asphalt, tarmac, or paved routes [10].

Scientists’ research has focused mainly on analysing multiple performance variables in UT such as body composition [11], biomechanics [12], or the physiological adaptations of these athletes [13]. However, due to the extreme characteristics of these races, researchers have also assessed the possible negative effects of these races on runners‘ health [14]. Some of the research carried out involving these races has revealed some medical conditions including different organs, such as the kidneys [15], heart, circulatory system [16], bones [17] and haematological alterations [18], but muscle damage (MD) stands out above the rest [19]. MD is assessed directly by blood damage biomarkers (creatine kinase (CK), lactate dehydrogenase (LDH)) [20] or by the presence of myoglobin in the urine (myoglobinuria) [21]. This muscle breakdown originates from the lack of energy in muscle cells or by the dysregulation in sodium–potassium channels [22]. MD may result in exertional rhabdomyolysis (ER) under some circumstances, including high-intensity exercise [23], lack of specific physical training in the type of exercise [24], extreme temperature and humidity (especially overheating), high altitude [25], and over- or dehydration, both during and after exercise [26]. ER is a medical condition, whereby skeletal MD is induced by excessive physical activity [22]. It can cause weakness and/or myalgia and may be frequently underdiagnosed in UT because of these confusing and overlapping symptoms [27]. Concerning the quantitative values to diagnose ER, there is no consensus in the scientific community. On some occasions, a reference value of 1000 U·L^−1^ to 10,000 UL^−1^ is used [28], while other protocols endorse the use of a 5-times elevation of normal CK upper limit values to define this condition [29]. CK levels tend to rise in the first 12 h, peak on the second or third day, and return to baseline 3–5 days or even 9 days after completing the race [30]. Traditionally, the presence of myoglobin in the blood was used to diagnose ER; however, it can result in misdiagnosis because of the similarity of this molecule with haemoglobin [31]. Because of this fact, the diagnosis of ER is complex, and the physician must have a high index of suspicion to accurately diagnose ER, with only the classic triad symptoms (myalgia, weakness, and myoglobinuria) being observed in only >10% of subjects eventually diagnosed with ER [32]. Due to this difficult diagnosis, other biomarkers have been proposed to help diagnose ER, including liver damage biomarkers such as aspartate transaminase (AST) and alanine aminotransferase (ALT) [33]. In 1978, Schiff et al. [34] already found some ER cases in a marathon. These episodes included a marked rise in AST and ALT despite no acute liver damage being diagnosed, and these values returned to baseline levels. The aminotransferases (AST and ALT) are involved in liver gluconeogenesis and can be found in multiple tissues, including the kidney, liver and muscle, and it is not easy to elucidate whether these alterations are due to liver damage or muscle injury [35]

The incidence of ER reported in previous studies ranges from 0% [36] to 43.5% [37], and it is very heterogeneous depending on the characteristics of the sport analysed. Rojas et al. [37] stated in a recent systematic review that 67.2% of the diagnosed cases of ER corresponded to UT runners compared to other ultra-endurance sports. Other sports such as CrossFit [24] and plyometric exercise [38] have reported similar ER incidence, both involving a high eccentric load on the athletes. These findings have provoked new investigations to establish a relation between concentric, isometric, and eccentric contractions, and MD. Royer et al. [39] found greater MD and muscle soreness after eccentric compared to concentric exercise, with these negative effects lasting longer in eccentric exercise. Considering these facts, the completion of UT downhill running has been proposed as a key factor in developing ER, MD, and muscle soreness [40]. In this systematic review, we examined the eccentric load that characterises the different UTs and the subsequent increases in the MD biomarkers CK, LDH, AST, and ALT to assess the apparition of ER episodes.

## 2. Materials and Methods

A systematic search of the relevant literature was conducted following the PRISMA method [41]. Four databases were thoroughly examined (PUBMED, WOS, Scopus, and SPORTdiscus), with a deadline of 10 March 2024. Of the terms used, a combination of 103 terms were predefined. The systematic review was accepted by PROSPERO (CRD 42024529946) on 8 April 2024. A combination of glossaries or thesauri was used: (mountain race) OR (ultra trail) OR (ultra-endurance race) OR (endurance race) AND (muscle damage) OR (rhabdomyolysis) OR (muscle damage biomarkers). For the full search equation, see Appendix A.

### 2.1. Criteria for Study and Selection

#### 2.1.1. Inclusion Criteria

- Adult population of both sexes;
- UT races (>42,195 km) and negative elevation of at least 1000 m;
- Muscle damage biomarkers included CK, LDH, MB, and/or liver damage biomarkers AST and ALT.

#### 2.1.2. Exclusion Criteria

- Population with previous medical conditions related to muscle illness;
- ER was not assessed;
- Combined sports (triathlon or biathlon);
- Another language different to English;

During the review process, the following information was collected from each of the studies: year of publication; author; participant-related data (number, mean age, and sex); type of event (distance, elevation gain, and number of stages); ER biomarkers (CK, pre or post, and increases or decreases), LDH (pre, post, and variations), AST (basal and post-race values, variations after the race), ALT (basal and post-race values, variations after the race), and ER diagnosis and treatment if required.

### 2.2. Overall Quality of the Studies

**Figure 1:**
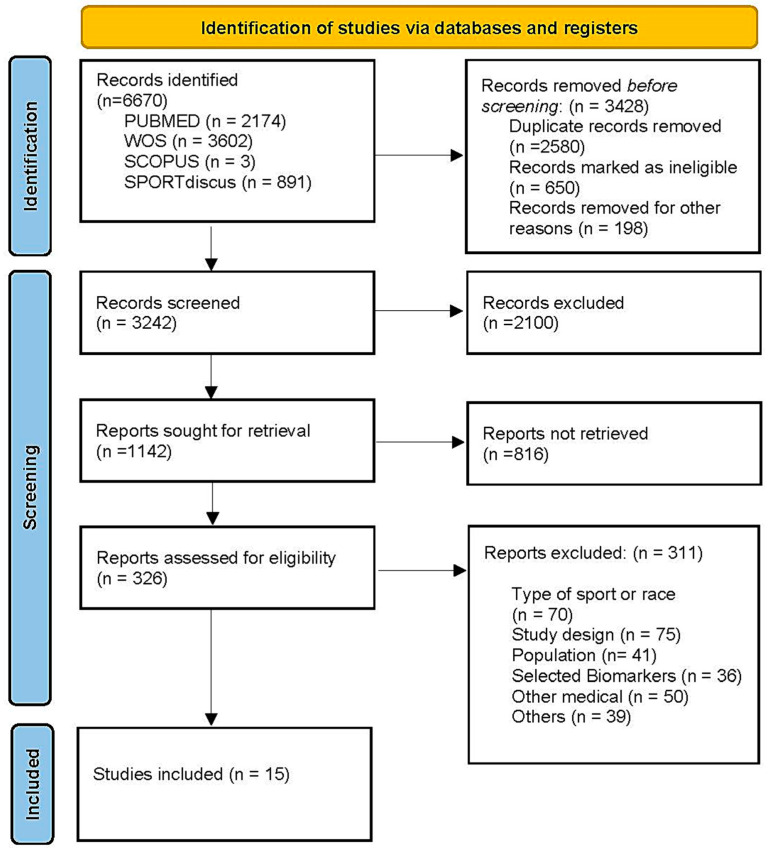
Flow chart describing the selection process of the included studies.

Two independent reviewers selected the included papers based on established criteria. Mendeley Desktop^®^ v.2112.0 (Elsevier, Amsterdam, The Netherlands) was used to remove duplicate articles and analyse both titles and abstracts. When necessary, a further full-text analysis was conducted. Decisions were always approved by both reviewers. However, in case of doubt or discrepancy, a third reviewer was consulted to solve the disagreement. The research and analysis process lasted a total of three weeks. The procedure followed in the review is described in detail in the flowchart (Figure 1).

### 2.3. Risk of Bias

Episodes of ER were assessed in prospective descriptive, cohort, and comparative studies, but no articles meeting the inclusion criteria for trial- or intervention-type designs in UT were found. The designs of the studies included descriptive studies not assessable with methodologically validated tools such as PEDro [41] or Cochrane Collaboration’s tool [42], which are commonly accepted as the gold standard. The tools used for quality assessment of observational descriptive studies specify that their use should be solely illustrative and only offer qualitative criteria [43].

Despite this, it was decided that the degree of evidence of the included studies should be evaluated using two tools. Firstly, the Quality Assessment Tool for Observational Cohort and Cross-Sectional Studies of the National Heart, Lung and Blood Institute (NHLB) was used [44]. This scale has 14 items that can be responded to with yes, no, Na (not applicable), Nr (not reported), or Cd (cannot be determined) and rates studies as good, fair, or poor. All 14 items were evaluated for each article. The tool does not use the number of “yes” answers to establish each of the categories, but rather leaves the decision of defining cutoff values for each category up to the user. Articles were classified as good when 11 or more items were marked “yes”, fair when this number was 7 to 10 items, and poor when only 6 or fewer items were marked “yes”. Secondly, the Rosenbrand et al. [45] tool for the level of evidence was used, where studies are evaluated with four letters (A, B, C, or D).

## 3. Results

The initial search of the literature identified 6670 potentially relevant articles. Following a review of titles and abstracts and excluding duplicates, the total was reduced to 326 articles. Of these articles, only 15 fully met the selection criteria and were finally included in this systematic review (Figure 1).

**Figure 2:**
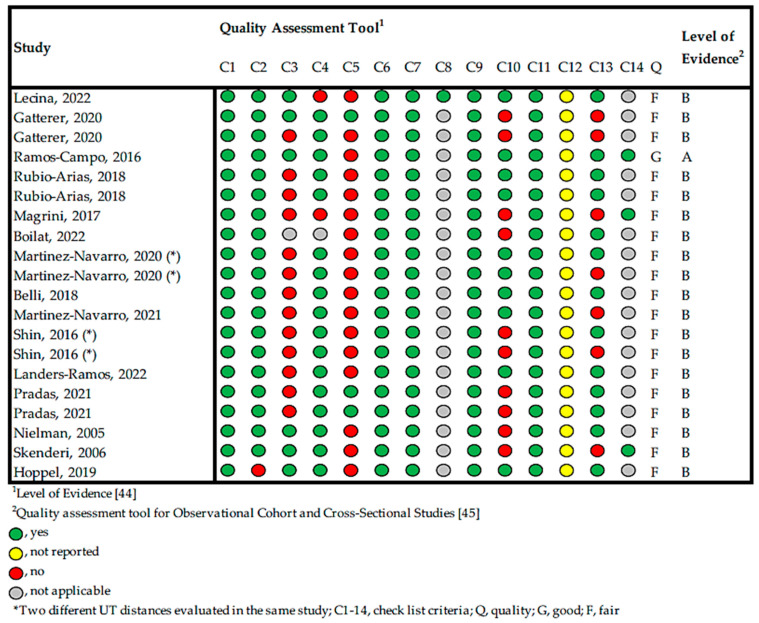
Risk of bias assessment.

Risk of bias assessment (Figure 2) showed that one of the studies included in the systematic review was classified as “good” or “A”, with a score of 11 out of 14 according to the NHLB [44] and Rosenbrand et al. [45], respectively. Regarding the nineteen remaining studies, they were classified as “fair” or “B”, obtaining scores ranging from 7 to 10 out of 14 [44,45].

The results were divided into three sections. The first one, “Types of UT and participant data”, discusses the characteristics of the UT analysed (duration, negative and positive elevation, number of stages, negative relative elevation, and population included); the second section, “ER biomarkers”, is organised according to the muscle damage biomarkers and the alterations in their values after completing the race; and finally, the section “liver damage” discusses the alterations found in liver enzymes (AST and ALT).

### 3.1. Types of UT and Participant Data

Fifteen studies were finally included in the systematic review [25,36,40,46,47,48,49,50,51,52,53,54,55,56,57]; five of them were comparative, including two different UTs [40,47,51,52,54], and were analysed as two different studies. The systematic review´s complete results are presented in Table 1. The total population sample included 348 subjects distributed as follows: 294 men (84.48%) and 54 women (15.52%). The age of the population was given as the mean and sd (39.82 ± 6.89 years). Only four of the twenty UTs analysed included highly trained populations [46,49,54,57], while the rest of the UTs studied [25,36,40,47,48,50,51,52,53,54,55,56] included amateur runners. As for the total number of subjects in each category, they were distributed as follows: 21 (6.03%) were highly trained runners and 327 were amateurs (93.96%), all of them being male athletes.

In terms of the type of races included (20), 2 were multi-stage [46,49], 18 were single-stage, 12 were extra category [25,36,40,47,50,51,52,54,55,57] and 6 were medium category [47,48,51,53,56] (Table 2). The negative elevation gains of the races were (median and sd) 4069.22 ± 2643.11, and the positive elevation gain was (median and sd) 4280.27 ± 2849.61. To facilitate the study, each race´s average negative elevation gain was calculated according to its duration in kilometres and the average negative elevation gain in meters. In the multistage UT [46,49], the mean level per stage was calculated to normalize these results. The mean value obtained was (median and sd) 35.97 ± 20.73 (Table 3).

### 3.2. ER and Biomarkers

Regarding ER episodes, only one study reported cases of ER with a clinical diagnosis that did not require hospitalization or medical treatment [46], so the incidence of ER in this systematic review was 0.28%. The biomarkers used in each of the included articles were, in order of greatest use: CK, analysed in all fifteen studies [25,36,46,47,48,49,52,54,55,56,57], followed by LDH, studied in only six articles [36,46,47,48,52,57].

As for post vs. pre CK increases, the results, expressed as mean and sd, were 5370.63 ± 7289.71 (295.24–11,507.94). The diagnostic criteria for ER diagnostics were met in 11 studies [25,36,46,52,54,55,56,57] and the increase in post vs. pre-CK was fivefold lower than baseline in only 3 [47,48,49]. Regarding the increase in the post vs. pre LDH values, only six studies assessed the LDH biomarkers [36,46,47,48,52,57]. The mean increase in LDH post vs. pre was (mean and sd) 311.20 ± 164.40.

### 3.3. Liver Damage

Liver damage related to MD was analysed using AST and ALT biomarkers and expressed as the median and standard deviation. AST was determined in five of the twenty races [36,46,52,57], obtaining a mean increase of 1009.94 ± 743.97%. ALT was analysed in only three studies [36,52,54], and the increase obtained was 207.02 ± 92.84%. No case of acute or chronic liver damage was reported, and consequently, no subject required medical treatment or hospitalization.

Independently of the distance and elevation loss covered, all the included studies (20 in total) reported augmentations in serum CK concentrations, ranging from 102.09 to 24,585.39% compared to baseline values. Those analysing serum LDH circulating levels (8 studies) observed a post-exercise increment of 175.62–597.59% from pre-exercise values. AST and ALT followed a similar pattern when comparing baseline to post-UT values in all 5 (+267.37–2385.71%) and 7 (+147.83–371.43%) studies where these biomarkers were assessed, respectively. 

## 4. Discussion

This systematic review aimed to analyse the eccentric load that characterized the different UT and the subsequent increases in MD biomarkers CK, LDH, AST, and ALT to assess the apparition of ER episodes. To the best of the authors´ knowledge, this has been the first systematic review analysing ER and the subsequent alterations in muscle (CK and LDH) and liver (AST and ALT) damage specifically in UT races. Comparing the aforementioned muscle and liver damage biomarkers studied here to previously published data, larger increases were found when comparing pre vs. post values of those reported in other endurance sports [37], novel sport exercises, and non-sports related exertion [58,59].

The internal load that occurs when completing an UT provokes larger increases in CK and LDH than in road and flat races [34,60]. These findings have led recent research to look for those UT characteristics responsible for alterations in MD biomarkers such as CK and LDH. Traditionally, duration, elevation gain and loss, as well as self-nutrition and self-hydration have been the key points of these kinds of races [2,10]. At the moment, several eccentric load in-lab studies have been carried out [61], but, to the authors’ knowledge, no studies have investigated outdoors UT or the effects of its long durations and eccentric load on muscle health in a systematic review specifically focused on UT.

### 4.1. Exertional Rhabdomyolysis

Only one subject was reported with ER in our systematic review (0.28%), a slightly lower incidence than the reported by Bäcker et al. (2.1%) in patients from various sports [58] and by (1–2%) in the US Army [62]. Nevertheless, these data may be underestimated due to a lack of protocols to evaluate ER and the previously reported overlapping symptoms of weakness and myalgia in addition to muscle swelling. In any case, those diagnosed with ER should require further investigation to determine the extent of their condition, if an acute kidney injury (AKI) [63,64] episode is linked to it, and if a higher level care is needed [65,66]. 

Looking at serum CK, it has been reported that exercise-related increases in CK are usually detectable soon after the end of the effort, reaching the peak around 24 h afterwards, and values return to baseline levels within 3–6 days [67]. It is unclear which is the best option to define ER through CK given that various methods have been defined: a 5-times elevation of normal CK upper limit values [29], a reference value of 1000 to 10,000 U·L^−1^ [28], or a substantial rise of >50,000 U·L^−1^ [32]. Although just one subject was reported with ER in the included publications, we subsequently identified non-diagnosed ER in all nine studies which reported post-UT CK values following Cleary et al.’s cut points [28]. Five of them [47,48,49,52,54] were within the defined limits (1000–10,000 U·L^−1^), and four [25,36,55,57] were well above the upper limit, with values ranging from 14,500 to 43,762 U·L^−1^. The eccentric load consequence of downhill running distributed in the type of UT was analysed as follows: single-stage [25,36,40,47,48,50,51,52,53,54,55,56] ranged from 315 to 12,100 m, and in the multi-stage, the negative elevation loss ranged from 4267 to 600-m [46,49]. This could be one of the main reasons for it [68], although no relationship between the amount of elevation loss and a subsequent, directly proportional increase in CK has been found. Furthermore, the possible influence of heat stress and dehydration in ER development should be taken into account, data which were not available in the included studies [69].

It is well documented that following muscle damage, plasma protein myoglobin also increases rapidly and is cleared quickly through renal excretion, and a normal level is re-established within 24 h [29]. Two of the included studies [48,57] assessed pre- and post-plasma myoglobin concentrations, and both reported increases of 95.30-fold (54 km; 2665-m), 67.71-fold (111 km; 4420-m), and 85-fold (54 km; 2665-m) baseline levels, respectively. It should be noted that when a large amount of muscle is injured, this protein is accumulated in the kidney, where it can increase the risk of developing AKI [28,37,66].

Another commonly used muscle damage biomarker is LDH, an enzyme which starts to be released into the bloodstream after 1–3 h of low- to moderate-intensity exercise, reaching its peak between 3 and 6 h afterwards and typically returning to baseline levels within 24 h [70,71]. In this review, only six studies [36,46,47,48,52,57] of fifteen evaluated LDH serum levels. All of them reported increases ranging from 1.76- to 5.98-fold pre vs. post levels. In these cases, LDH augments were accompanied by a 2.61–245.85-fold increase in CK concentrations, which reinforces the impact of UT on athletes’ muscle health.

After developing and suffering from ER, return to normal activity is recommended to be gradual, increasing in extent and duration if serum CK levels have normalised and associated symptoms have disappeared. Furthermore, exercise involving eccentric loads and strenuous activities should be avoided at the beginning [68]. The acute treatment includes hydration, ice, massage, and painkillers and may even require hospitalization [20,29]. Hydration plays a vital role in the development ER, not only due to dehydration, but also over-hydration. Fluid replacement during UT has been proven as a risk factor and is a pathogenic factor for developing exercise-associated hyponatremy (EAH). EAH increases intracellular fluid and, as a consequence, generates weakness of the myocyte cell membranes and promotes their rupture and ER, releasing myoglobin and other elements into the bloodstream [70]. Eventually, it may cause the accumulation of some of these myoglobins in the tubular cells [72].

They were heading to a rapid influx of calcium ions into muscle cells. This fact can trigger a pathophysiological cascade, resulting in a pathological interaction between actin and myosin and activation of cell protease with subsequent necrosis of muscle fibres, as well as the release of intracellular metabolites (potassium, phosphates, and urates) and intracellular proteins (myoglobin, creatine kinase, aldolase, lactate dehydrogenase, among others) in the extracellular space and bloodstream. In the case of a single event (i.e., mostly trauma), CK levels tend to rise in the first 12 h, peak on the second or third day, and return to baseline 3–5 days later.

### 4.2. Eccentric Load

This systematic review aimed to determine whether greater negative elevations in UT lead to larger muscle damage biomarkers and an increased risk of ER episodes, either alone or with AKI. We followed the PRISMA method [41] and conducted a quantitative analysis of the UTs included (Table 1). We ranked them based on their duration and negative elevation loss to examine whether there was a direct relationship between the two factors (Table 2 and Table 3). Our hypothesis was not fully supported, as UTs with higher elevations did not consistently result in larger CK increases. We considered a meta-analysis, but the included studies were descriptive, and there were too few that met the inclusion criteria to carry out the analysis.

There were studies with high negative relation, such as Belli et al. [57] and Pradas et al. [54], where the increases in CK parameters were larger than the average CK increase (2207.77% and 14141.67%, respectively). However, studies such as Skenderi et al. [36], which included the lowest ratio between duration and elevation, showed the highest increase in CK (24585.39%). This heterogeneity in results can be explained by the variety of factors affecting this type of race. This situation has been repeated in many other systematic reviews which analysed ER and related medical conditions in ultra-endurance sports such as AKI and ER [26,37], AKI and EAH [30], AKI alone [15], ER alone in the adult population [58], or ER in adolescent athletes [73], all of them concluding that ER episodes are the result of multifactorial risk factors, but it is impossible to determine the specific weight of every one of them.

The eccentric contraction has been extensively studied in laboratory tests and intervention studies [74,75]. In a laboratory test, Hody et al. [71] analysed CK increases, muscle soreness, and stiffness after 30 min of running on a treadmill with an eccentric load. They found that higher increases in the three parameters occurred as long as the negative elevation was increased. Paddon et al. [76] analysed the role of speed along with eccentric load as a pathogenic in a comparative study on the role of speed along with eccentric load as a pathogenic factor of ER. CK increases were similar in slow and fast groups, and both showed CK increases of more than fivefold when comparing post vs. pre.

Other studies have compared concentric and eccentric exercise. Margaritelis et al. [77] compared eccentric exercise versus concentric exercise after completing 75 min of running on a treadmill. Eccentric exercise showed larger CK increases; however, after ten weeks of training, the results were similar between both groups. These results have been supported by Pradas et al. [54], who studied muscle damage in a UT by comparing two groups with different levels of training. No statistical differences were found between the groups when comparing CK increases. With similar results, Coratella et al. [78] analysed the CK increases after 30 min downhill running at 10 km·h^−1^ and −20% for four days in a row. CK increases were larger on the first day in comparison with the fourth day. According to these results, training and accumulating a sufficient eccentric load may be vital in preventing runners from suffering ER episodes, demonstrating the importance of a coherent training process when it comes to facing these extreme races.

### 4.3. Liver Damage

This systematic review found increases in both liver damage biomarkers, AST and ALT. Both enzymes have been traditionally evaluated in ER as complementary diagnostic biomarkers [20,79]. AST was evaluated in four studies [36,46,52,57] and showed an increase, with pre values versus post values of 1009.94 ± 743.97%, whereas ALT was only analysed in three studies [36,52,54], and the variation in post values versus pre values was smaller (207.02 ± 92.82%). These findings align with the other studies where these biomarkers have been analysed in exercise, especially in ultra-endurance sports [80]. Several studies have found increases in both enzymes after heavy muscular exercise associated with ER [35]. AST is present in cytosolic and mitochondrial isoenzymes and is found in the liver, cardiac muscle, skeletal muscle, kidneys, brain, pancreas, lungs, leucocytes, and red cells [81]. Contrary, ALT is more concentrated in liver tissue than in muscle tissue; this fact results in higher specificity for diagnosing liver damage than analysing AST [82]. The release of these aminotransferases to blood torrent is also different; AST is usually detectable at 24 h, while ALT may still be in the normal range until 48 h. AST tends to peak around 3 to 4 days, and the ALT peaks later at 4 to 5 days, but some studies in UT have shown larger alterations, such as Lecina et al. [46], where these values did not return to baseline nine days after completing the UT.

The still-unanswered question concerns up to which point these alterations imply real liver damage or whether they are mere transitory alterations. Contrary to ER, acute liver failure episodes have scarcely been reported in studies analysing liver damage in UT [60]. De Castro et al. [80] found liver failure in a non-professional runner who completed a 10 km race, but the cause was a heat stroke, and no ER was associated with this case. Liver damage in endurance sports has been associated with ER or other medical conditions that are sometimes not diagnosed in this context [14]. Pal et al. [83] found some ER episodes in 44 post-pubertal boys and girls who underwent intensive treadmill exercise. They also demonstrated that serum ALT and AST increased significantly at 24 and 48 h in association with raised CK and LDH. Sometimes runners use non-steroidal drugs to alleviate the pain that these races inflict on their bodies, and the effect of the use of these drugs, when non-prescribed, may lead to liver damage after completing the race or even days after [84]. In light of these results, an association between ER and liver damage seems unlikely, and when fatal damage is present, it is due to underlying causes which are underdiagnosed.

- Limitations

This systematic review only considered studies in English, which may result in language bias. Additionally, the diagnosis of ER relies on analysing various biomarkers for MD, and there is no consensus in the scientific community regarding its diagnosis, particularly in healthy populations. Furthermore, randomized studies with a control group are not feasible in UT races, making meta-analysis impossible. Consequently, the lack of this type of analysis greatly limits the extent to which conclusions can be drawn. However, by following the PRISMA guidelines, this systematic review’s internal validity is guaranteed, and it was successfully registered in Prospero. Finally, there is a potential risk of bias due to the variety of UT races and the confusion surrounding their classification and definition. Despite the inclusion of criteria such as a minimum distance of 42 km and natural environments, the races still varied significantly in distance, as demonstrated in Table 1, Table 2 and Table 3. 

## 5. Conclusions

In conclusion, UT races increase the MD biomarkers CK and LDH. Excessive eccentric and unaccustomed exercise are among the significant risk factors for experiencing an ER episode. However, it is challenging to establish universal criteria for diagnosing ER and estimating the risk factors associated with completing a UT. Although ER episodes are typically self-limited and rarely require medical treatment in the context of UT, circumstances such as dehydration, lack of proper training, extreme temperature, and the use of some medications can cause AKI and pose a risk to runners’ health. Eccentric exercise significantly increased MD biomarker levels. However, our systematic review indicates that UT race risks cannot be categorized solely based on elevation loss. More studies are required in order to establish specific criteria for diagnosing ER and prevent runners from suffering ER episodes, ultimately avoiding further complications such as AKI.

## Figures and Tables

**Table 1:** Included studies demonstrating subsequent ER after completing a UT.

Author; Year	Population; Number; Sex; Age	Race Length; Stages; Elevation +/−	CK Pre, Post & Rec	LDH Pre, Post, and Rec	AST Pre, Post, and Rec	ALT Pre, Post, and Rec	ER Cases (%)
Lecina, 2022 [46]	Highly trained; 4; 0 F, 4 M; 38.0 ± 4.11 yrs	786 km; 11; 46,865+ m; 46,845− m;	↑CK Pre 98.51 ± 24.53 Post 974 ± 402.66 R2 474.85 ± 185.70 ↓CK R9 88.00 ± 16.27	↑LDH Pre 172.75 ± 14.71 Post 470.30 ± 104.80 R2 316 ± 70.88 R9 208 ± 31.55	↑AST Pre 23.75 ± 3.20 Post 63.50 ± 9.68 R2 44.50 ± 8.74 R9 48.25 ± 27.45	↑ALT Pre 17.25 ± 3.59 Post 44.75 ± 12.44 R2 37.25 ± 8.80 R9 56.25 ± 34.25	1 (25%)
Gatterer, 2020 (*) [40]	Amateur; 11; 0 F, 11 M; 42.0 ± 9.0 yrs	68 km; 1; 4260+ m; 4260− m	↑CK Post 1562 ± 1250	-	-	-	0 (0%)
Gatterer, 2020 (*) [40]	Amateur; 7; 0 F, 7 M; 41.0 ± 10.0 yrs	121 km; 1; 7554+ m; 7554− m	↑CK Post 4933 ± 3760	-	-	-	0 (0%)
Ramos-Campo, 2016 [48]	Amateur; 11; 0 F, 11 M; 29.7 ± 10.2 yrs	54 km; 1; 2726+ m; 2665− m	↑CK Pre 820 ± 2087.3 Post 2421.1 ± 2336.2	↑LDH Pre 383 ± 178.6 Post 795 ± 260.7	-	-	0 (0%)
Rubio-Arias, 2018 (*) [47]	Amateur; 10; 0 F, 10 M; 27.0 ± 5.7 yrs	54 km; 1; 2726+ m; 2665− m	↑CK Pre 886.6 ± 2187.9 Post 2213.8 ± 2354.5 R1 1014 ± 732.6 ↓CK R2 495.8 ± 358 R3 309.4 ± 191.6	↑LDH Pre 406.9 ± 135 Post 731.3 ± 161.1 R1 449.8 ± 66.9 R2 430.9 ± 72.1 R3 409.9 ± 59.8	-	-	0 (0%)
Rubio-Arias, 2018 (*) [47]	Amateur; 6; 0 F, 6 M; 30.5 ± 8 yrs	111 km; 1; 4474+ m; 4420− m	↑CK Pre 174 ± 197.4 Post 8976 ± 4327.1 R1 2132.5 ± 1399.6 R2 1277.7 ± 1368.2 R3 604.2 ± 878.3	↑LDH Pre 335.1 ± 35.2 Post 751 ± 57.2 R1 588.7 ± 90.7 R2 551.1 ± 72.7 R3 509.1 ± 56.7	↑LDH Pre 335.1 ± 35.2 Post 751 ± 57.2 R1 588.7 ± 90.7 R2 551.1 ± 72.7 R3 509.1 ± 56.7	-	0 (0%)
Magrini, 2017 [25]	Amateur; 36; 8 F, 28 M; 43 yrs (26–68)	160 km; 1; 4800+ m; 4800− m	↑CK Pre 126 ± 64 Post 14,569 ± 14,729	-	-	-	0 (0%)
Boillat, 2022 [49]	Highly trained; 1; 0 F, 1 M; 34 yrs	619 km; 7; 600+ m; 1100− m	↑CK Inc post vs. pre (102%)	-	-	-	0 (0%)
Martínez-Navarro, 2020 (*) [51]	Amateur; 17; 4 F, 13 M; 41 ± 7 yrs	65 km; 1; 4200+ m; 4200− m	↑CK Pre-Post Pre-R1	↑LDH Pre-Post Pre-R1	-	-	0 (0%)
Martínez-Navarro, 2020 (*) [51]	Amateur; 32; 13 F, 19 M; 41 ± 6 yrs	107.4 km; 1; 5604+ m; 4356− m	↑CK Pre-Post Pre-R1	↑LDH Pre-Post Pre-R1	-	-	0 (0%)
Belli, 2018 [57]	Highly trained; 6; 0 F, 6 M; 47 ± 5 yrs	217 km; 1; 12,200+ m; 12,200− m	↑CK Pre 132 ± 18 84 k 3988 ± 1004 177 k 18,667 ± 10,664 Post 19,157 ± 12,369	↑LDH Pre 371 ± 66 84k 677 ± 80 177k 2026 ± 671 Post 1986 ± 687	↑AST Pre 28 ± 3 84k 109 ± 13 177k 677 ± 406 Post 668 ± 407	-	0 (0%)
Martínez-Navarro, 2021 [50]	Amateur; 34; 5 F, 29 M; 39 ± 7 yrs	118 km; 1; 5439+ m; 4227− m	↑CK Pre-Post Pre-R3	↑LDH Pre-Post Pre-R3 Pre-R7	-	-	0 (0%)
Shin, 2016 (*) [52]	Amateur; 17; 0 F, 17 M; 48.35 ± 3.14 yrs	100 km; 1; 1000+ m; 1000− m	↑CK Pre 132.76 ± 55.17 Post 2983.1 ± 715.56	↑LDH Pre 338.82 ± 97.02 Post 693.58 ± 323.56	↑AST Pre 26.35 ± 14.02 Post 126.58 ± 35.58	↑ALT Pre 23.88 ± 18.05 Post 43.64 ± 32.98	0 (0%)
Shin, 2016 (*) [52]	Amateur; 16; 0 F, 16 M; 51.43 ± 2.89 yrs	308 km; 1; 1000+ m; 1000− m	↑CK Pre 131.75 ± 39.34 Post 4970.31 ± 2222.48	↑LDH Pre 385.62 ± 57.55 Post 1002.31 ± 224.60	↑AST Pre 25.87 ± 7.33 Post 203.50 ± 99.70	↑ALT Pre 21.75 ± 6.01 Post 78.06 ± 30.76	0 (0%)
Landers-Ramos, 2022 [53]	Amateur; 11; 3 F, 8 M; 40 ± 7 yrs	50 km; 1; 762+ m 762− m	↑CK Pre-10km Pre-Post Pre-R1	-	-	-	0 (0%)
Pradas, 2021 (*) [54]	Amateur; 10; 0 F, 10 M; 43.3 ± 4.52 yrs	108 km; 1; 5800+ m 5800− m	↑CK Pre 164.3 ± 69.39 Post 3251.6 ± 1011.89	-	-	↑ALT Pre 18.6 ± 2.5 Post 31.7 ± 9.67	0 (0%)
Pradas, 2021 (*) [54]	Highly trained; 10; 0 F, 10 M; 41.4 ± 6.18 yrs	108 km; 1; 5800+ m 5800− m	↑CK Pre 193.6 ± 42.11 Post 4261.5 ± 1469.6	-	-	↑ALT Pre 23.5 ± 3.92 Post 37.1 ± 7.46	0 (0%)
Nieman, 2005 [55]	Amateur; 60; 15 F, 45 M; 45.3 ± 1.1 yrs	160 km; 1; 5500+ m 6700− m	↑CK Pre 159 ± 21 Post 17,833 ± 2883	-	-		0 (0%)
Skenderi, 2006 [36]	Amateur; 39; N/A; 41 ± 1 yrs	246 km; 1; 1200+ m 1200− m	-	-	-	-	0 (0%)
Hoppel, 2019 [56]	Amateur; 8; 0 F, 8 M; 41.5 yrs	67 km; 1; 2500+ m; 2500− m	-	-	-	-	0 (0%)

(*): comparative studies including two UT races; F: female; M: male; yrs: years; km: kilometres; m: meters of elevation; +: slope positive; −: slope negative; ±: positive and negative elevation mixed; ↑CK: elevation in creatine kinase (UI/L) from baseline; ↓CK: decrease in creatine kinase from baseline (UI/L^−1^); ↑LDH: elevation in lactodeshydrogenase (UI/L) from baseline; ↑AST: elevation in aspartate aminotransaminase (UI/L) from baseline; ↑ALT: elevation in alanine aminotransferase (UI/L) from baseline; ↑MYO: elevation in myoglobin (μg/L) from baseline; ↑CRP: elevation in c-reactive protein (mg/L) from baseline; = CRP: maintenance in c-reactive protein (mg/L) from baseline; -: not reported or not available; Pre: baseline; 84 k: 84 km in-race; 100 k: 100 km in-race; 150 k: 150 km in-race; 177 k: 177 km in-race; Pre: immediately before race; Post: immediately after race; Rec: recovery period immediately after the end of the race; R1: 24 h recovery after the race; R2: 48 h recovery days after the race; R3: 72 h recovery days after the race; R7: 7 recovery days after the race; R9: 9 recovery days after the race.

**Table 2:** UT race classification according to distance, elevation gain, elevation loss, and duration/elevation loss ratio.

UT Race Classification	Distance (km) (Range)	Elevation Gain (m) (Range)	Elevation Loss (m) (Range)	Negative Relative Elevation Loss (Range)
Medium	(50–60)	(762–4260+)	(762–4260−)	(15.24–64.62)
Extra	(100–308)	(1200–12,200+)	(1200–12,200−)	(1.02–62.43)
Multi-stage	(619–786)	(600–46,865+)	(1100–46,865−)	(1.78–59.62)

+: positive elevation gain; −: negative elevation loss.

**Table 3:** Included studies and the relationship between duration (km) and negative elevation (m).

Author; Year; Reference	Rank	Negative Relative Elevation Loss
Martínez-Navarro, 2020 (*) [50]	1	64.62%
Gatterer, 2020 (*) [40]	2	62.65%
Gatterer, 2020 (*) [40]	3	62.43%
Belli, 2018 [51]	4	56.22%
Pradas, 2021 (*) [55]	5	53.70%
Pradas, 2021 (*) [55]	6	53.70%
Rubio-Arias, 2019 (*) [48]	7	49.35%
Ramos Campo, 2016 [47]	8	49.35%
Nieman, 2005 [56]	9	41.88%
Martínez-Navarro, 2020 (*) [50]	10	40.71%
Rubio Arias, 2019 (*) [48]	11	39.82%
Martínez-Navarro, 2021 [52]	12	35.82%
Magrini, 2017 [25]	13	30.00%
Hoppel, 2019 [57]	14	29.85%
Shin, 2016 (*) [53]	15	21.00%
Landers-Ramos, 2022 [54]	16	15.24%
Lecina 2022 [46]	17	5.42%
Skenderi, 2006 [36]	18	4.88%
Boillat, 2022 [49]	19	1.78%
Shin, 2016 (*) [53]	20	1.00%

(*): comparative studies including two UT races. Negative elevation loss: duration of the race in km/negative elevation loss expressed in meters.

## Data Availability

The data will be made available by the corresponding author upon reasonable request.

## Appendix A

(((((mountain race) OR (ultra trail)) OR (ultra endurance race)) OR (endurance race)) AND (muscle damage)) OR (rhabdomyolysis)) OR (muscle damage biomarkers),Most Recent,”Clinical Trial, Meta-Analysis, Randomized Controlled Trial, Review, Systematic Review, Humans, English”,”(((((““mountain”“[All Fields] OR ““mountaineer”“[All Fields] OR ““mountaineering”“[MeSH Terms] OR ““mountaineering”“[All Fields] OR ““mountaineers”“[All Fields] OR ““mountainous”“[All Fields] OR ““mountains”“[All Fields]) AND (““racial groups”“[MeSH Terms] OR (““racial”“[All Fields] AND ““groups”“[All Fields]) OR ““racial groups”“[All Fields] OR ““race”“[All Fields])) OR (““ultra”“[All Fields] AND (““trail”“[All Fields] OR ““trail s”“[All Fields] OR ““trails”“[All Fields])) OR (““ultra”“[All Fields] AND (““endurance”“[All Fields] OR ““endurances”“[All Fields]) AND (““racial groups”“[MeSH Terms] OR (““racial”“[All Fields] AND ““groups”“[All Fields]) OR ““racial groups”“[All Fields] OR ““race”“[All Fields])) OR ((““endurance”“[All Fields] OR ““endurances”“[All Fields]) AND (““racial groups”“[MeSH Terms] OR (““racial”“[All Fields] AND ““groups”“[All Fields]) OR ““racial groups”“[All Fields] OR ““race”“[All Fields]))) AND ((““muscle s”“[All Fields] OR ““muscles”“[MeSH Terms] OR ““muscles”“[All Fields] OR ““muscle”“[All Fields]) AND (““damage”“[All Fields] OR ““damaged”“[All Fields] OR ““damages”“[All Fields] OR ““damaging”“[All Fields]))) OR (““rhabdomyolysis”“[MeSH Terms] OR ““rhabdomyolysis”“[All Fields] OR ““rhabdomyolyses”“[All Fields]) OR ((““muscle s”“[All Fields] OR ““muscles”“[MeSH Terms] OR ““muscles”“[All Fields] OR ““muscle”“[All Fields]) AND (““damage”“[All Fields] OR ““damaged”“[All Fields] OR ““damages”“[All Fields] OR ““damaging”“[All Fields]) AND (““biomarker s”“[All Fields] OR ““biomarkers”“[MeSH Terms] OR ““biomarkers”“[All Fields] OR ““biomarker”“[All Fields]))) AND ((clinicaltrial[Filter] OR meta-analysis[Filter] OR randomizedcontrolledtrial[Filter] OR review[Filter] OR systematicreview[Filter]) AND (humans[Filter]) AND (english[Filter]))
